# Aquatic Macrophytes Occurrence in Mediterranean Farm Ponds: Preliminary Investigations in North-Western Sicily (Italy)

**DOI:** 10.3390/plants10071292

**Published:** 2021-06-25

**Authors:** Patrizia Panzeca, Angelo Troia, Paolo Madonia

**Affiliations:** 1 Dipartimento di Scienze e Tecnologie Biologiche, Chimiche e Farmaceutiche, Università Degli Studi di Palermo, 90123 Palermo, Italy; patriziapanzeca@gmail.com; 2 Istituto Nazionale di Geofisica e Vulcanologia, Sezione di Roma 2, 00143 Roma, Italy; paolo.madonia@ingv.it

**Keywords:** Mediterranean flora, aquatic flora, carbon isotopes, hydrophytes, conservation, bioindicators, inland waters, water geochemistry

## Abstract

Mediterranean wetlands are severely affected by habitat degradation and related loss of biodiversity. In this scenario, the wide number of artificial farm ponds can play a significant role in the biodiversity conservation of aquatic flora. In the present contribution we show the preliminary results of a study on Mediterranean farm ponds of north-western Sicily (Italy), aimed to investigating the environmental factors linked to the occurrence of submerged macrophytes (vascular plants and charophytes). We studied the aquatic flora of 30 ponds and determined the chemical and isotopic composition of their water bodies on a subset of the most representative 10 sites. Results show that (1) farm ponds host few but interesting species, such as *Potamogeton pusillus* considered threatened at regional level; (2) *Chara vulgaris, C. globularis* and *P. pusillus* behave as disturbance-tolerant species, occurring both in nitrates-poor and nitrates-rich waters, whereas *Stuckenia pectinata* and *Zannichellia palustris* occur only in nitrates-poor waters. Although farm ponds are artificial and relatively poor habitats, these environments seem to be important for the aquatic flora and for the conservation of the local biodiversity, and can give useful information for the use of macrophytes as bioindicators in the Mediterranean area.

## 1. Introduction

Mediterranean wetlands are severely affected by habitat degradation and related loss of biodiversity [1,2], and for this reason they are protected by various international (EU) and national laws. In spite of this, the knowledge about flora of these wetlands is poor [2], making difficult their proper management.

Sicily is a hotspot of plant biodiversity [3], hosting a plenty of habitats including wetlands (such as saltpans, temporary ponds, coastal ponds, small lakes, etc.), even if a lot of them have been destroyed in recent times, and especially in the last two centuries. The Sicilian landscape is characterized by the occurrence of a wide number of farm ponds, which are supposed to play a significant role for the life of aquatic species, and this is the reason why we focused our attention on their flora, presently poorly investigated.

Farm ponds are usually considered artificial poor habitats, although it has been shown that sometimes they host a significant biodiversity, contributing to maintain rich trophic webs, from producers to consumers and decomposers [4,5,6,7]. Although biodiversity of these habitats is usually poorer than that of natural ones [8,9], it has been shown that the two groups are rather comparable as far as hydrophytes and red-listed species is concerned [9].

In the present contribution, we show the first results of a study aimed at individuating the environmental variables linked to the occurrence of aquatic macrophytes (vascular plants and charophytes) in Mediterranean farm ponds of north-western Sicily, with particular attention to the chemical and isotopic characteristics of their waters. Numerous studies have shown the importance of aquatic macrophytes as a key component of lake ecosystems, as they provide refuge and food for various organisms, influence the nutrient availability in water, and enhance the stability of lake shores ([10] and literature cited therein). In detail, some recent studies focused on the possibility of using aquatic macrophytes as bioindicators [11,12], or of testing the effects of high concentrations of nitrates or phosphates in the water (e.g., [13]), but we made special reference to the work of Gallego et al. [14] on the macrophytes in Mediterranean farm ponds.

Our results will be discussed not only with reference to biodiversity and conservation issues, but also to the EU Water Framework Directive 2000/60/EC [15] and to the need of assessing the quality of inland waters through biotic indicators.

## 2. Study Area Settings

**Figure 1:**
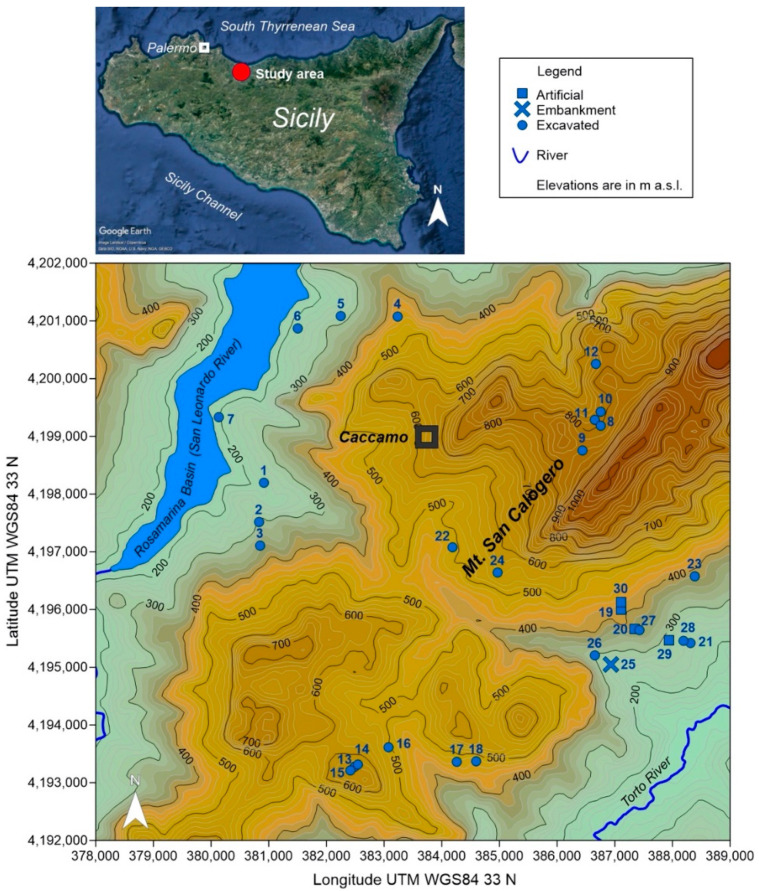
Location of the studied area (at the upper left) and map showing position and constructive characteristics of the ponds.

The studied area is located in the western sector of the coastal northern mountainous chain of Sicily (Italy), close to the town of Caccamo, about 35 km SE of the regional capital city of Palermo (Figure 1). It is comprised in the altitudinal belt 200–900 m a.s.l., characterized by a clayey hill landscape, dominated by the SW-NE oriented carbonate massif of Mt. San Calogero (1326 m a.s.l.) and punctuated by sparse outcrops of Mesozoic gypsum (Gessoso-Solfifera Formation). To NW the area is delimited by the Rosamarina basin, created by blocking with a dam the San Leonardo River; its SE limit is the Torto River.

**Figure 2:**
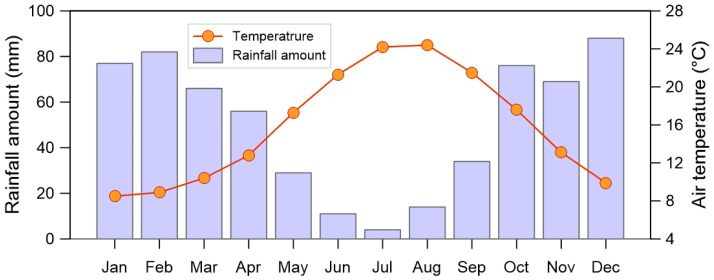
Average (1965–1994) monthly air temperature and rainfall amount; data are from the Ciminna station, the closest available to the study area showing comparable orographic conditions [16].

The climate is typical of the Mediterranean area, with mild-humid winters alternating to hot-dry summers. Figure 2 reports the average (1965–1994) monthly air temperatures and rainfall amounts measured at Ciminna, the closest available meteorological station whose orographic location (elevation and distance from the coastline) is similar to that of the investigated area [16]. Minima of temperature (8 °C) are recorded in January, while the hottest months are July and August, with average values over 24 °C. The dry season spans from May to September; the driest month is July, with few millimetres of rain on average.

Land use is characterized by traditional semi-intensive agriculture with a mosaic of cultivated and uncultivated land.

## 3. Materials and Methods

Pond selection followed criteria of geo-lithologic representativeness, as well as pond construction-management typologies [14]. Since their construction and management are closely related in our study area, ponds were classified according to these criteria. Artificial ponds (A) are small-sized ponds made by concrete or masonry, which are (usually) intensively managed, showing high water renewal rates and scarce littoral vegetation cover (helophytes and/or riparian vegetation). Excavated ponds (E) exhibit a natural substrate, high water renewal rates, high coverage of littoral vegetation, and are moderately managed. Embankment ponds (D) are obtained by blocking small streams: they have a natural substrate, with a continuous renewal ensured by the flowing water, and rarely managed.

A total of 30 ponds were selected for this study, whose location and characteristics are reported in Figure 1 and Table 1. Their surface areas range from a minimum of 2 m^2^ to a maximum of 2395 m^2^, and their altitudes from 193 to 838 m a.s.l. Only mountain ponds (8–12) fall within a protected area, the Monte San Calogero Regional Natural Reserve, which is also a Natura2000 site (code ITA020033) according to the EU 92/43/CEE “Habitats” Directive. Chemical and isotopic analyses on the stored water bodies were carried out on a subset of 10 sites, representative of the different managing and construction criteria, geo-climatic conditions and water origins. Sites pertaining to this subset are highlighted in bold in Table 1.

A single sampling session was carried out on 20 November 2019. Electric conductivity, pH and Eh of water were measured in the field, using electrochemical sensors. Water clarity was measured in situ with a Secchi disc. Samples for the determination of dissolved major and trace elements (Table 1), taken close to the free water surface, were first filtered using 0.45 µm Millipore MF filter and then collected in LD-PE bottles for major element analyses, acidifying with HNO_3_ to ca. pH 2 the aliquot destined to cation determination. Untreated aliquots were collected for isotopic and alkalinity determinations, made via titration with HCl (0.1 N).

Water samples were analysed at the lab facilities of Istituto Nazionale di Geofisica e Vulcanologia (INGV), Sezione di Palermo. Major ions were determined by ionic chromatography, using Dionex columns AS14 and CS12 for anions and cations respectively. The determination of δ^18^O [17] of water was performed by CO_2_-water equilibration technique using a Thermo Delta V Plus instrument, equipped with a Gas Bench II. The results were reported in δ‰ versus the V-SMOW standard, with a precision better than ±0.1‰.

We focused our study to the strictly aquatic macrophytes, corresponding to the hydrophytes of the Raunkiaer’s system [18], including submersed, floating-leaved, and free-floating species, and not including the emergent ones (“helophytes”, as in [10]). The floristic survey was performed between June and November 2019: all the 30 ponds were sampled a first time between June and July, and a second time between October and November. Macrophytes were collected from the shores, using a grapnel or a rake. The abundance of macrophytes was evaluated, on sight from the shore, using a 5-rank qualitative estimation scale: 1 = very rare; 2 = infrequent; 3 = common; 4 = frequent; 5 = abundant/predominant. Every pond was considered as a single sampling unit. Since our ponds were relatively shallow and small, we are confident that our observations on composition and abundance of aquatic macrophytes, made from the shores, represent composition and abundance of the whole pond. After washing the fresh material to remove sediments and organic matter, fresh (charophytes) or dried (angiosperms) were observed using a stereomicroscope (Leica MZ9.5, maximum magnifications of 60×). Characeae were identified and named following Mouronval et al. (2015) [19], vascular plants were identified and named after Pignatti et al. (2017–2019) [20].

Principal component and correlation analyses were performed using PAST software (version 3.26 [21]). Kendall’s tau test was used to verify the correlation between measured parameters (including macrophytes occurrence). Kendall’s correlation is a nonparametric measure of the strength of the associations between two variables, which can be also used in the analysis of correlations between quantitative and ordinal parameters in case of a small number of observations. Then, statistical significance was tested, and *p*-values were determined.

## 4. Results

### 4.1. Physical and Chemical Characterization of the Ponds

**Figure 3:**
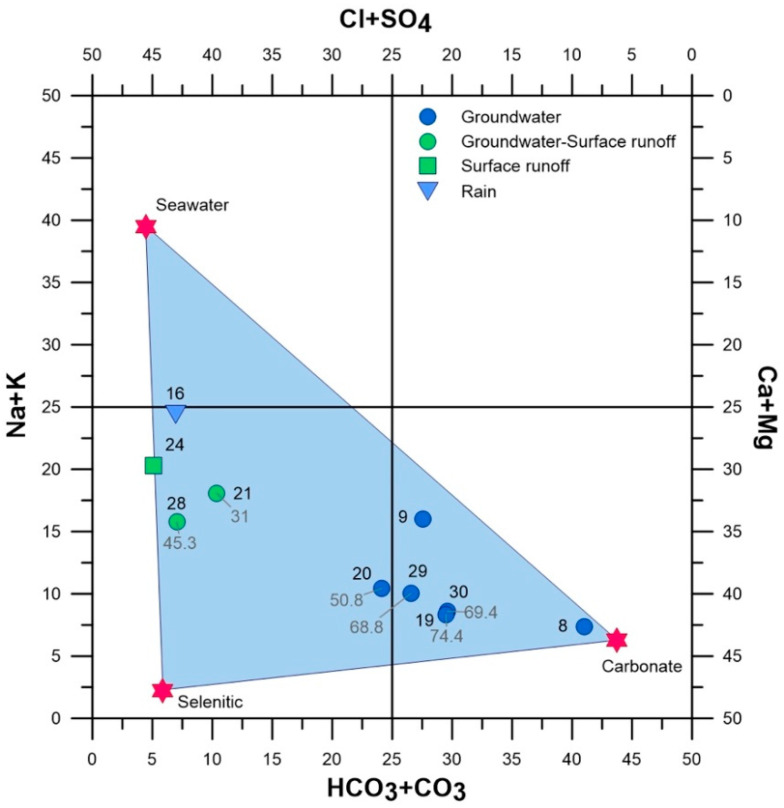
Langelier–Ludwig diagram showing the chemical facies of the analysed waters (numbers of the sites according to Table 1). Grey labels associated to sample points indicate the concentration of nitrates, expressed in mg L^−^^1^.

Chemical and isotopic data in the selected subset are presented in Table 2. A graphical representation of the chemical composition of water accumulated in the ponds, based on the Langelier–Ludwig diagram [22] is given in Figure 3. The diagram describes the geochemical facies of a natural water considering the concentrations (expressed in meq L^−1^) of two couples of main cations and two of anions, and reporting to 50 the sums of anions and cations, respectively.

The analysed water samples fall in a triangular area delimited by the compositions typical of three end members: seawater, carbonate and selenitic waters. This distribution reflects the characteristics of the major sources of dissolved solids. Groundwater circulates into aquifers hosted in limestones and dolostones outcropping at Mt. San Calogero (Figure 1), and water-rock interactions are responsible of the carbonate nature of these waters, whose best representation is found in site 8. This pure carbonate character is progressively modified by the chemical interactions with the atmospheric particulate, also composed by sea spray (upper vertex of the triangular area) and gypsum particles (lower left vertex), which are sources of alkali, chlorine and sulphates.

The dissolution of air particulate, both suspended in the atmosphere or deposited on the ground and leached by the flowing water, gives the chemical imprinting to sites 16, 21, 24 and 28, fed by surface runoff and direct rain, and less dependent on groundwater contributions.

These different feeding sources are also reflected by the concentrations of nitrates (Figure 3, grey labels), which in sites 19, 29 and 30, and in 20 at a lesser extent, are over the important threshold of 50 mg L^−1^ (on this topic see [23]). The higher concentrations are found in ponds fed by groundwater, indicating that nitrates, coming from fertilizers dispersed on the ground, are leached by infiltrating rainwaters and successively delivered to groundwater bodies.

**Figure 4:**
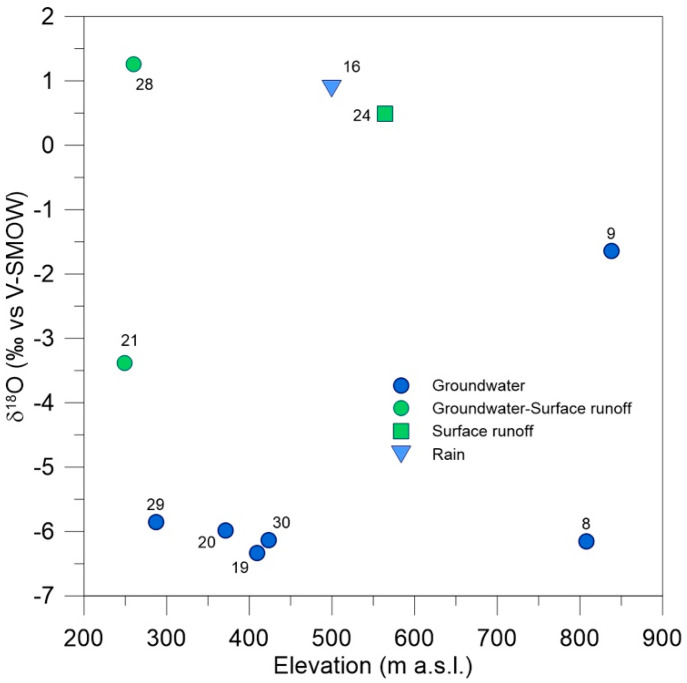
Vertical oxygen isotopic gradient of water accumulated into the studied ponds (numbers of the sites according to Table 1).

Other information, useful for identifying the main sources feeding the ponds, are given by the plot illustrating the variation with altitude of the oxygen isotopic compositions of the pond water (Figure 4).

Points representative of ponds fed by groundwater (ponds number 8, 19, 20, 29 and 30) show small vertical variations of δ^18^O: only few decimals of per mil around an average value of −6‰, which is congruent with local reference values for groundwater bodies [24]. The sole exception is pond 9: although it is fed by groundwater, the volume of water accumulated inside is so small to be strongly affected by evaporation, thus explaining the shift towards positive values. Values close or over 0‰, typical of surface evaporated water at these latitudes, are shown by sites 9, 16, 24 and 28, confirming that the provenience of water is more linked to surface runoff and/or direct rain; site 21 falls in an intermediate position between the above-described groups. These different positive isotopic shifts remark that evaporation have occurred at different extents, controlled by different factors. Ponds 9, 24 and 28 have low depths and large surface areas, which are conditions fostering direct evaporation, and consequently a positive isotopic shift. An additional influencing factor is the inter-time between two consecutive water extractions from the ponds (renewal time): the longer it is, the most the water will be affected by evaporation.

**Figure 5:**
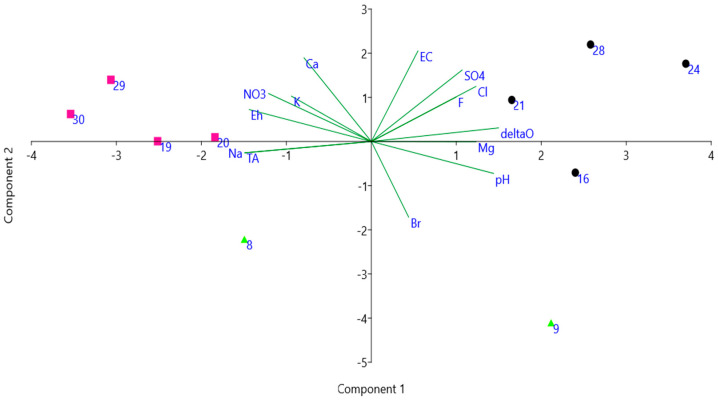
PCA of data shown in Table 2, excluding temperature and macrophytes occurrence. Abbreviations as in Table 2. Pink squares—ponds rich in NO_3_, black circles—ponds rich in SO_4_, green triangles—ponds with low values of EC (numbers according to Table 1).

A principal component analysis has been performed on the data shown in Table 2 (excluding temperatures and macrophytes occurrence), using a correlation matrix since variables are measured in different units (Figure 5). PCA axis 1 explained 53.395% of the total variance, with dδ^18^O, pH, SO_4_ and Cl showing positive loadings and Na, T.A., Eh and NO_3_ showing negative loadings. PCA axis 2 explained 26.217% of the total variance, with EC showing positive loading and Br showing negative loading. According to the analysis above, we found sites 19, 20, 29, 30 on the left side of the diagram, representing ponds fed by groundwater and rich in NO_3_; sites 16, 21, 24, 28 on the right part of the diagram, representing ponds fed by surface runoff and/or direct rain, and rich in SO_4_; ponds 8 and 9 fall in the lower part of the diagram, including (mountain) ponds with low values of EC.

### 4.2. Flora of the Ponds

In 22 out of 30 ponds one aquatic macrophyte at least was retrieved; minimum occurrence was zero, maximum two, with a median of one species. A total of five species was found in the ponds: two charophytes (*Chara vulgaris* L. and *C. globularis* Thuill.) and three angiosperms (*Potamogeton pusillus* L., *Stuckenia pectinata* (L.) Börner, *Zannichellia palustris* L.). All of them are rooted submerged plants; no rooted-floating or free-floating species were found. If one species occurs in a pond, it is a charophyte or an angiosperm, but if two species are present, one is a charophyte and the other one is an angiosperm (we never found two different angiosperms or two different charophytes in the same pond). *Potamogeton pusillus* is a species rare in Sicily, according to the regional red-list [25] where it is listed as vulnerable. No alien aquatic macrophytes were found.

**Figure 6:**
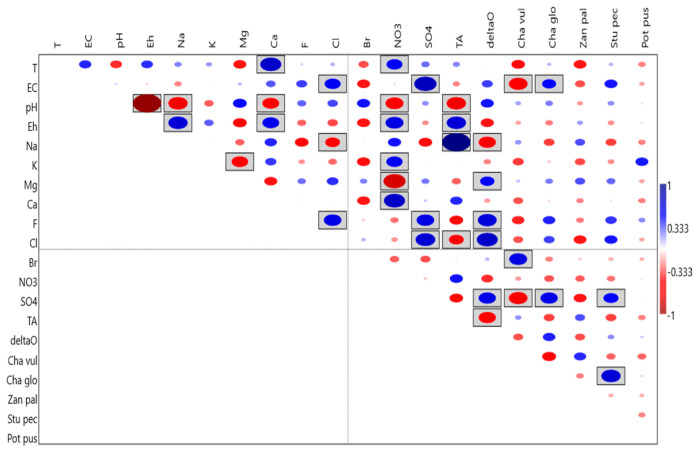
Correlation analysis using Kendall’s tau test; *p* < 0.05 are boxed. Abbreviations as in Table 1.

The results of the correlation analysis (using Kendall’s tau test) are shown in Figure 6. Regarding the correlations between different macrophytes, there is a significant positive correlation between *Stuckenia pectinata* and *Chara globularis*. Both charophytes show a significant correlation with EC and SO_4_, which is positive for *Chara globularis* and negative for *C. vulgaris*. *Stuckenia pectinata* shows a significant positive correlation with SO_4_.

**Figure 7:**
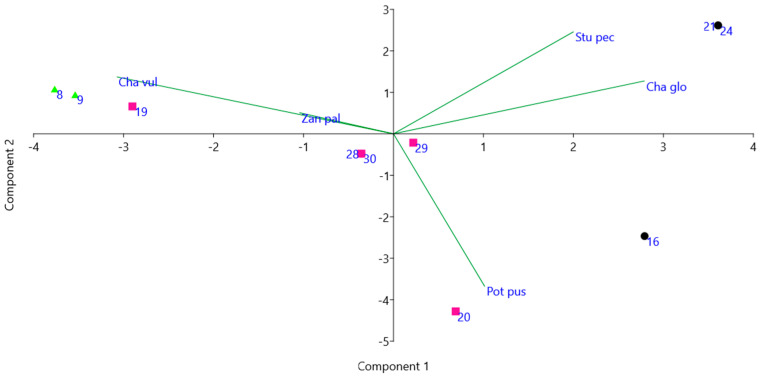
PCA of macrophytes’ occurrences shown in Table 2. Pink squares—ponds rich in NO3, black circles—ponds rich in SO4, green triangles—ponds with low values of EC (numbers according to Table 1).

Correlation between *S. pectinata* and *C. globularis* is also evidenced by PCA made on macrophytes’ occurrences (Table 2 and Figure 7); we used a var–covar matrix because variables are measured in the same units. PCA axis 1 explained 48.053% of the total variance, axis 2 28.321%; *C. vulgaris* and *Z. palustris* fall in the same quarter, but without a significant correlation, as shown in Figure 6. The studied ponds occupy different areas of the diagram illustrated in Figure 7, partially reproducing the groups based on chemical and physical parameters evidenced in Figure 5: ponds 8 and 9 are on the left side, not far from pond 19, ponds 16, 21 and 24 are on the right side, pond 28 is coincident with pond 30, while ponds rich in NO_3_ are scattered.

## 5. Discussion

### 5.1. Role of Man-Made Farm Ponds for the Biodiversity of the Agricultural Landscape

In Europe, traditional agriculture has created landscapes of considerable conservation value both from ecological and cultural points of view [26,27]. In this context, farm ponds have received considerable attention because of their critical role for the conservation of biodiversity in agricultural landscapes [28,29,30,31,32,33].

Our preliminary analysis has shown that farm ponds can contribute to increase the overall biodiversity of the agricultural landscape, hosting species not present otherwise. Ponds also act as a refuge station for the rare *Potamogeton pusillus*, included in the Red List of the vascular flora of Sicily. Submerged hydrophyte meadows represent a feeding source and a shelter for the wild fauna, and play a role in water purification [10].

In the framework of the European “Habitats” Directive, although only mountain ponds (sites 8–12) fall within a Natura2000 site (code ITA020033), in the ponds whose bottom is covered totally or partially by charophytes we can recognize a habitat of community interest, i.e., “Hard oligo—mesotrophic waters with benthic vegetation of *Chara* spp.” (code 3140).

The number of hydrophytes we found in the pilot area is comparable with other similar contexts (see for example [34]). However, a “rich” pond of our study hosts one charophyte and one angiosperm. Conversely, the Rebuttone gorge near Palermo hosts at least three charophytes and two angiosperms [35], and in the well preserved mountain ponds of the Nebrodi Mountains up to ten vascular hydrophytes (apart from charophytes) can be found in a single pond [36].

*Chara globularis* is found together with *Stuckenia pectinata* in six ponds, associated to *Potamogeton pusillus* in one site. In both cases, in phytosociological terms, the two co-occurring species do not constitute an association, as—although they are present in the same pond-they constitute two (physically and ecologically) well-separated monospecific communities, to be referred to two different phytosociological classes: *S. pectinata* and *P. pusillus* are to be referred to the class *Potametea pectinati* while *C. globularis* is to be attributed to the class *Charetea intermediae* (for the relationships between the two classes see [35]).

### 5.2. Use of Aquatic Macrophytes for Monitoring the Quality of Inland Waters in the Mediterranean Area

Data here reported refer to a survey limited both in space and time, not taking into account either seasonal variations or the role of macrophytes on biogeochemical cycle, as those of nitrogen and phosphorus. Nevertheless, their usefulness is remarkable, considering the scarcity of information about the aquatic flora of artificial ponds of the central Mediterranean region, and of Sicily in particular. According to the Water Framework Directive [15], species composition and abundance of macrophytes are biological components to be used in the assessment of the ecological quality of freshwaters. In this scenario, even if the Directive excludes the monitoring of lakes and reservoirs with surfaces <0.2 Km^2^ and <0.5 Km^2^, respectively, our preliminary data will be useful in testing the application of ecological synthetic indexes, with particular reference to macrophyte indexes suitable for Mediterranean farm ponds.

Phosphates are absent, except in the ponds 29 and 30, where they are present with high concentrations, while nitrate concentrations are high in ponds 29, 30, 19 and 20, with the last two fed by 30. Nitrates remain below 50 mg L^−1^ in ponds 28 and 21, which are fed by 29 entirely or partially, respectively. Nitrates are absent in the other ponds. Based on nitrate concentrations, ponds can be divided in two different groups:

- Ponds entirely or partially fed by groundwater, rich in nitrates (29, 30, 19, 20) or with values close to the eutrophication threshold (28 and 21); the sole exception is site 8, located in the poorly anthropized area of Monte San Calogero, where nitrates are absent.
- Ponds fed by surface waters, characterized by low nitrate concentrations (24, 16 and 9).

With the exceptions of pond 19, where *Chara vulgaris* is present, and pond 29, where scattered specimens of *C. globularis* have been found, in both cases associated to more or less abundant filamentous algae, the two charophytes are associated with non-eutrophic waters, confirming that the presence of *C. vulgaris* and *C. globularis* indicates a ‘good’ ecological status of the waters in shallow lakes [10,37].

*Stuckenia pectinata* and *Potamogeton pusillus* often grow in co-presence with charophytes that, as just discussed, are indicators of a good ecological status, but in our study area they are absent if nitrates are present in high concentrations. It is well known that *S. pectinata*, commonly associated with eutrophic hard waters, occurs even at low concentrations of nutrients [38]. Our findings then confirm that *S. pectinata* and *P. pusillus* are not exclusive of eutrophic waters, but should be considered disturbing-tolerant and growing in both oligotrophic and eutrophic waters.

*Zannichellia palustris* occurs in a single pond (associated to *C. vulgaris*), whose water is poor in nitrates: according to the literature it is another disturbing-tolerant species like the previous ones [37].

## 6. Conclusions

Although preliminary, our study showed that an artificial habitat such as a farm pond can be strategically important for the surviving of aquatic species (plants and charophytes, in this case): here in fact, we found a red-listed angiosperm, and a habitat of community interest. In addition, the presence of those (even few) species can be linked to the quality of the water and in general to the quality of the environment, supplying useful data for the development of ecological indexes that can be applied in artificial and natural ponds of the Mediterranean region.

## Figures and Tables

**Table 1:** Sampled sites (in bold those selected for geochemical analyses); geographic coordinates are in decimal degrees WGS84, elevations in m a.s.l., areas in m^2^. “Water” indicates the feeding source: surface run-off (S), direct rain (R), groundwater (G). “Type” is the construction method: excavation (E), concrete or masonry (A), stream barrage (D). “Use” is the utilization: irrigation (IRR), cattle watering (CWA), drinking trough (DTR) and not used (NUS). “Depth”: we separated two groups, “S” (shallow ponds, depth 0.5–1.5 m) and “D” (deep ponds, depth 2–8 m). Visibility: we separated two groups, “C” (clear waters) and “T” (turbid waters). The field “SM” reports the number of submerged macrophytes found.

Id	Longitude	Latitude	Elevation	Area	Water	Type	Use	Depth	Visibility	SM
1	13.6451	37.9235	224	638	R-S	E	IRR	D	C	2
2	13.6442	37.9174	309	93	R	E	CWA	D	C	2
3	13.6445	37.9137	349	24	R	E	CWA	S	C	1
4	13.6710	37.9498	438	379	R	E	NUS	D	T	0
5	13.6598	37.9497	280	408	R	E	NUS	D	T	2
6	13.6513	37.9477	220	224	R-S	E	CWA	D	C	1
7	13.6360	37.9337	193	623	R	E	CWA	D	C	1
**8**	**13.7114**	**37.9332**	**808**	**343**	**G**	**E**	**CWA**	**S**	**C**	**2**
**9**	**13.7078**	**37.9293**	**838**	**114**	**G**	**E**	**CWA**	**S**	**C**	**1**
10	13.7113	37.9354	786	66	G-R	E	CWA	S	C	2
11	13.7102	37.9341	785	48	G-R	E	CWA	S	C	2
12	13.7103	37.9428	767	213	R	E	CWA	D	T	0
13	13.6638	37.8794	641	42	R	E	CWA	D	T	1
14	13.6644	37.8798	628	113	R	E	NUS	D	T	0
15	13.6630	37.8788	649	43	R	E	CWA	D	T	1
**16**	**13.6704**	**37.8825**	**500**	**129**	**R**	**E**	**IRR**	**D**	**C**	**2**
17	13.6914	37.9100	472	273	R	E	NUS	D	T	0
18	13.6877	37.8805	469	422	R	E	NUS	D	T	0
**19**	**13.7159**	**37.9045**	**409**	**64**	**G**	**A**	**IRR**	**D**	**C**	**1**
**20**	**13.7186**	**37.9015**	**371**	**16**	**G**	**A**	**NUS**	**S**	**C**	**1**
**21**	**13.7297**	**37.8995**	**249**	**527**	**G-S**	**E**	**IRR**	**D**	**C**	**2**
22	13.6825	37.9139	486	1034	S	E	IRR	D	C	1
23	13.7303	37.9099	387	401	R	E	IRR	D	T	2
**24**	**13.6914**	**37.9100**	**564**	**2395**	**S**	**E**	**IRR**	**D**	**C**	**2**
25	13.7140	37.8959	255	54	S	D	NUS	S	C	1
26	13.7108	37.8973	296	208	S	E	IRR	S	C	1
27	13.7195	37.9014	359	233	G	E	IRR	D	T	0
**28**	**13.7283**	**37.8998**	**260**	**891**	**G**	**E**	**IRR**	**D**	**T**	**0**
**29**	**13.7254**	**37.8998**	**287**	**2**	**G**	**A**	**DRT**	**S**	**C**	**1**
**30**	**13.7159**	**37.9057**	**423**	**4**	**G**	**A**	**DRT**	**S**	**C**	**0**

**Table 2:** Physical, chemical and isotopic data of the water stored in the pond subset selected for analyses, including presence of macrophytes in each pond. Temperature (T) in °C, electric conductivity (EC) in μS cm^−1^, Eh in mV, dissolved ions in meq L^−1^ (except NO_3_^−^ and PO_4_^3−^ in mg L^−1^), δ^18^O in ‰ V-SMOW, b.d.l. is below detection limit (0.01 meq L^−1^). Abbreviations for macrophytes species (whose abundance was evaluated using a 5-degree estimation scale, see the text): Cha vul (*Chara vulgaris*), Cha glo (*Chara globularis*), Zan pal (*Zannichellia palustris*), Stu pec (*Stuckenia pectinata*), Pot pus (*Potamogeton pusillus*).

Id	8	9	16	19	20	21	24	28	29	30
T	10.9	12.8	13.1	15.4	14.0	16.2	14.3	15.6	19.3	18.5
EC	572	452	834	805	720	1012	1363	1152	1052	832
pH	7.51	8.59	8.40	7.80	7.51	8.02	8.31	8.03	7.00	6.98
Eh	−50	−108	−98	−66	−50	−77	−93	−78	−23	−22
Na^+^	5.40	2.72	1.15	4.86	3.47	2.32	1.62	1.73	5.87	5.03
K^+^	0.09	0.06	0.16	0.22	0.24	0.11	0.14	0.13	0.16	0.22
Mg^2+^	2.24	2.14	1.59	0.66	0.71	1.56	3.87	2.07	1.07	0.66
Ca^2+^	3.14	1.24	2.70	6.97	5.56	5.73	5.54	6.77	8.75	7.15
F^−^	0.01	0.02	0.03	b.d.l.	0.03	0.07	0.03	0.09	0.02	b.d.l.
Cl^−^	1.05	1.84	1.73	1.29	1.34	2.93	3.23	3.27	1.78	1.33
Br^−^	b.d.l.	0.05	b.d.l.	b.d.l.	b.d.l.	b.d.l.	b.d.l.	b.d.l.	b.d.l.	b.d.l.
NO_3_^−^	b.d.l.	b.d.l.	b.d.l.	69.4	50.8	19.2	b.d.l.	45.3	68.8	74.4
SO_4_^2−^	0.13	0.37	5.42	2.06	2.39	5.95	11.0	7.28	3.39	2.17
PO_4_^3−^	b.d.l.	b.d.l.	b.d.l.	b.d.l.	b.d.l.	b.d.l.	b.d.l.	b.d.l.	4.75	0.95
CO_3_^2^^−^ + HCO_3_^−^	5.40	2.72	1.15	4.86	3.47	2.32	1.62	1.73	5.87	5.03
δ^18^O	−6.15	−1.64	0.88	−6.33	−5.98	−3.38	0.49	1.26	−5.85	−6.13
Cha vul	4	5		4						
Cha glo			4			4	4		1	
Zan pal	4									
Stu pec						4	4			
Pot pus			4		5					

## Data Availability

All relevant data are presented as tables or can be found in the cited references.
